# Managing and Mitigating Suffering in the Return-to-Work Process

**DOI:** 10.3389/fpsyg.2021.805855

**Published:** 2021-12-09

**Authors:** Megan Woods, Mandy L. Matthewson

**Affiliations:** ^1^Tasmanian School of Business and Economics, College of Business and Economics, University of Tasmania, Hobart, TAS, Australia; ^2^School of Psychological Sciences, College of Health and Medicine, University of Tasmania, Hobart, TAS, Australia

**Keywords:** return-to-work, suffering, injury, workplace health and safety, wellbeing, workers’ compensation

## Abstract

Each year thousands of workers experience a serious illness or injury that necessitates time off work and a subsequent re-engagement with the work environment. In Australia, workers’ compensation legislation mandates the return-to-work (RTW) process is formal, structured, and negotiated between the worker, their employer, health care professionals and their RTW coordinator. How this is executed by those parties directly influences whether the RTW process is supportive and successful, or exacerbates the suffering of returning workers by causing them to feel ostracised, exposed, and vulnerable in their workplace. In this article, we examine how the RTW process can cause physical, emotional, social, and existential suffering for returning workers. We then discuss how the suffering that workers experience can be mitigated by five key factors: clarity of roles in the RTW process, alignment of worker and employer expectations, the advocacy provided by the RTW coordinator, the support provided for the worker’s psychological wellbeing, and the RTW literacy of supervisors and colleagues.

## Introduction

Each year thousands of workers experience illness or injury that necessitates time off work and subsequent re-engagement with their work environment. Sickness-related absence from work has a range of negative impacts on workers and their families so returning to work is often perceived as the desired end to a painful life experience. This depends, however, on the return-to-work (RTW) process being responsive and supportive to the worker and their needs. When it is not, it can extend their experience of feeling different, exposed, and vulnerable because of their health situation. Greater understanding of the suffering that workers can experience during RTW is crucial to optimising the responsiveness, empathy and support workplaces provide. In this article we examine the physical, emotional, social, and existential suffering that can arise from the RTW process. We then examine how that suffering can be mitigated by five key factors: clarity of roles in the RTW process, alignment of worker and employer expectations, the advocacy provided by the RTW coordinator, support provided for the worker’s psychological wellbeing, and the RTW literacy of supervisors and colleagues.

## Suffering

Suffering occurs when “threat or damage to one’s body or self-identity” causes a person distress (Anderson, 2013, p. 10) or to feel diminished (Cassell, 2004). Physical suffering results from distress or diminishment related to one’s physical being (Anderson, 2013). In a RTW context, physical suffering can result from physical pain caused by the original injury/illness or from treatment (e.g., surgery), the physical effects of treatments (e.g., nausea caused by medications), or physical strain experienced when resuming work-related activities with impaired ability.

Mental suffering is distress or diminishment related to cognitive or affective self-identity (Anderson, 2013) and suffering caused by psychological ill-health (e.g., depression, anxiety, and post-traumatic stress disorder). It includes cognitive suffering caused by thoughts or thought processes (e.g., worries or anxieties) and suffering caused by emotional responses (Anderson, 2013). In a RTW context, mental suffering might relate to the illness/injury itself (such as fear and anxiety resulting from physical pain). It may also stem from the injury/illness’s impact on the person’s life and potential future, such as fears their ill-health may reduce their capacity to support themselves and their families.

Existential suffering occurs when people experience threats to their existence or struggle with the meaning of their life (Anderson, 2013). In a RTW context, existential suffering could occur when a person with a vocation or profession which comprises a large part of “who they are” is then unable, due to illness or injury, to do their work and be their professional self. For example, a medical practitioner whose illness precludes them from practicing medicine might question the meaning of their life if they cannot treat people anymore.

Social suffering is both a form of suffering and a way of understanding how social contexts shape and contribute to other forms of suffering. Indeed, (Cassell, 2004, p. 34) contends that all suffering is inherently social because “suffering exists and can only be understood in the context of others.” Social suffering arises from social sources and forces, including social institutions and cultures (Anderson, 2013) such as workplaces (Sanchez-Hernandez et al., 2020). In a RTW context, social suffering can be caused or mitigated by social norms, beliefs and practices relating to work, illness, and injury (Cassell, 2004), or by workplace policies and practices which “disable” people with impaired health (Woods et al., 2019). For example, a workplace culture framing work-related injuries as the fault of the injured workers could produce social suffering resulting from stigmatisation.

## Suffering in the Return-to-Work Process

Noordik et al. (2011) characterise the RTW process as a dynamic and iterative problem-solving process in which the worker interacts with RTW stakeholders and their work environment to develop and apply potential solutions to barriers to full return to work. However, the barriers and social dynamics that workers encounter are all potential sources of suffering.

### Suffering Experienced When Developing the Return-to-Work Plan

In Australia, workers’ compensation legislation mandates that the RTW process includes development of a RTW plan (WorkSafe Tasmania, 2021). When the returning worker is judged medically fit to return to work, a RTW plan is negotiated with input from a variety of RTW stakeholders. These stakeholders include the worker, their manager, insurer, and workers’ compensation representatives, the health professionals overseeing their care and the RTW coordinator (Corbiere et al., 2020). This process includes a formal assessment of the worker’s support needs, and the development of workplace accommodations which can include adjustments to duties, adaptations to physical work environments, and changes to working hours (Woods, 2012). The planning process needs to identify both the factors that the worker perceives will influence their RTW, and how these can be anticipated or overcome (Corbiere et al., 2017). However, achieving this is complicated by the suffering that has already resulted from the worker’s ill-health.

Illness, injury, and treatment regimens can all produce physical suffering from pain and strain, and mental suffering from fears and anxiety, such as fears about the severity of their health situation, and anxiety about pain (Standal et al., 2021). Mental and social suffering also arise from the loss of capacity to perform tasks and fulfil roles, and a change in social identity from that of a healthy, functioning person to that of a sick individual who is no longer functioning well (Standal et al., 2021). As negative impacts on the person’s physical and psychological functioning become pronounced, mental, and social suffering results from the attendant damage to their self-esteem and self-confidence, family relationships, and their capacity to perform roles in their social, family, personal, and work lives (Boden et al., 2001). This in turn can fuel mental suffering caused by fear, and stress about the financial impacts of their illness/injury, such as loss of earnings and being forced to use savings, borrow money, or draw on pensions or superannuation to meet their needs (Dembe, 2001). All these forms of suffering can reduce returning workers’ cognitive and emotional capacity to engage in RTW planning.

When the process of working through assessments and decisions in RTW planning highlights the worker’s loss of capacity it exacerbates mental and social suffering. Questions about how their health will negatively affect their pace and quality of work, or capacity to handle work responsibilities may fuel fears that they will be unable to work as before (Dembe, 2001), causing both mental suffering related to fears about their work-related future, and social suffering resulting from a diminution of their perceived value as an employee. How these assessments and decisions are handled can also cause mental and social suffering. Workers suffer when they feel “medically misunderstood” (MacEachen et al., 2007, p. 159) or “forced” back to work and, by extension, feeling unsupported, isolated, and treated with disrespect (Kirsh and McKee, 2003). For example, when their experience is doubted and disbelieved returning workers experience mental and social suffering arising from suspicion, damaged relationships, distrust, and feelings of powerlessness (MacEachen et al., 2007).

### Suffering Experienced During Re-entry to the Workplace

A returning worker re-entering their workplace can feel particularly vulnerable because workplace accommodations and supports publicly signal their loss of capacity to those around them. The appropriateness of work adaptations, and the sensitivity with which they are handled, directly influence the degree of social and mental suffering associated with re-entry into the work environment. They can also cause physical suffering when insufficient workplace accommodations, unrealistic rehabilitation expectations and pressure to resume pre-injury work duties and work pace create untenable working conditions (Gewurtz et al., 2018) causing pain, strain, and potential secondary injuries.

Additionally, the worker’s state of health may fluctuate as a function of their RTW experiences meaning the process may be prolonged and non-linear. Sometimes finding the “best fit” supports and accommodations for the individual is a process of trial and error, which can be correlated with changes in the employee’s health. This exacerbates mental and social suffering when it creates uncertainty about the individual ever being able to fully return to work, and/or overextends the sympathy and supportiveness of others in the workplace. When uncertainty surrounds the outcome of their injury/illness, this can also cause a loss of coherence in their professional identity, to themselves and with others. For example, if an agricultural worker can no longer perform that work, the loss of that professional identity can cause mental, social and even existential suffering when it causes questioning about “who they are now.” This is worsened when that work is a focal point of the local community and neighbours, friends, and community members are former workmates (MacEachen et al., 2007).

## Mitigating the Suffering That Workers Experience During the Return-to-Work Process

We examine below five factors that research evidence and our clinical experience of supporting RTW show are key to optimising RTW effectiveness and mitigating suffering for the returning worker.

### Clarity of Roles in the Return-to-Work Process

When the returning worker understands the roles, rights, and responsibilities they and other stakeholders have in the RTW process, they are able to better navigate and actively engage with that process (Dean et al., 2019). Role clarity is the extent of an individual’s understanding about their duties and expectations of their tasks and roles (Hinkin and Schriesheim, 2008). Role clarity can be improved when employees have the information they need to perform their duties; a sense of control over work tasks; supportive and encouraging leadership in the workplace; and clarity about the roles of others within the organisational structure (Kauppila, 2013). Good relationships and clear communication between the returning worker and other RTW stakeholders are critical for achieving role clarity.

Poorly defined or conflicting roles can exacerbate suffering during the RTW process by causing the returning worker to feel helpless and uncertain. Low role clarity can create confusion about and conflict between a worker’s responsibilities, causing mental suffering associated with anxiety, depression, burnout, and job dissatisfaction (Barnett et al., 2010). Optimising role clarity can therefore mitigate both mental and social suffering that results from feeling misunderstood, unsupported, and helpless. Further, when all parties are clear about the limits of each persons’ role, the individual is more likely to have realistic expectations of themselves and others involved in the RTW process. The RTW coordinator has an important role to play in this process. They can provide information and clarification on roles for all parties. They are also well placed to identify when lack of role clarity is causing suffering and problem solving needs to occur to mitigate suffering.

### Alignment of Expectations in Return-to-Work Process

Positive expectations about the RTW process are a critical factor in its success (Corbiere et al., 2017) and a strong predictor of a successful RTW (Opsahl et al., 2016). RTW expectations are influenced by worker perceptions about the obstacles they will encounter during RTW, and whether those obstacles can be overcome. These expectations also form the basis for the worker’s return-to-work self-efficacy (RTW-SE), which is “the belief that workers have in their ability to meet the demands of their job should they return to work” (Nieuwenhuijsen et al., 2013, p. 291). Low RTW-SE may result from overly negative or irrational perceptions of the RTW process but can also reflect a realistic evaluation of their work environment (Nieuwenhuijsen et al., 2013). It is therefore important that the returning worker’s RTW-SE be considered in the RTW plan. Practitioners working with the returning worker can help by assessing self-efficacy and helping the worker to identify whether their beliefs are associated with an unrealistic return to work plan or are indicative of ailing mental health.

Aligning expectations of the worker and other parties in the RTW process reduces mental suffering by providing more certainty and transparency about what is expected and how reintegration into the workplace will be progressively implemented and supported. This helps minimise mental suffering caused by fears of being expected to do too much too soon and physical suffering that could otherwise result from physical strain or secondary injury. By ensuring all stakeholders understand what the worker is capable of, and that suitable supports are in place, it can also improve understanding and empathy for the worker and minimise social suffering.

Alignment with expectations held by co-workers is also important. Modified duties for the returning worker can produce an increased workload for co-workers. This can lead to resentment and erosion of relationships in the workplace, undermine social connectedness and, in turn, negatively impact the returning worker’s psychological wellbeing (Dunstan and MacEachen, 2013; Kosny et al., 2013). This can be mitigated when co-workers are assigned clearly defined, time limited, mutually agreeable roles in the RTW plan (Dunstan and MacEachen, 2013; Kosny et al., 2013).

### Advocacy Provided by the Return-to-Work Coordinator

Return-to-work coordinators help plan and support the RTW process and may be employed at the worker’s organisation, employed by insurance or workers’ compensation entities, or be independent consultants (MacEachen et al., 2020). RTW coordinators have specialised knowledge of relevant policies, legal responsibilities and procedures, and can offer valuable process support by initiating the RTW process, providing timely information and support to supervisors (Lysaght and Larmour-Trode, 2008), convening regular meetings of the RTW team and acting as a conduit between team members. Research attests that RTW coordinators can shorten disability duration (Franche et al., 2005) and improve RTW rates (Dol et al., 2021) although there is emerging evidence these impacts may differ for RTW from psychological ill-health (MacEachen et al., 2020).

Return-to-work coordinators can reduce the power imbalance between the returning worker and other stakeholders in the process by providing both a voice and a buffer for the returning worker (MacEachen et al., 2007). They can advocate for the returning worker when the RTW process exceeds the returning worker’s limitations (Noordik et al., 2011), which is particularly important when the RTW plan is not well supported by the workplace. When RTW coordinators can help to ensure that accommodations, supports, and work plans appropriately reflect and support worker needs, they help to minimise physical, mental, and social suffering.

### Support for the Returning Worker’s Psychological Wellbeing

It has long been recognised that psychological and physical states are intrinsically linked (Kirsh and McKee, 2003). Workplace injury in particular can have a serious psychosocial impact on the injured worker when it results in feelings of anxiety, depression, stress, increased conflict in the home and with family (Dembe, 2001). Support for the returning worker’s psychological wellbeing is therefore crucial to minimising the negative impact on the worker of their experiences of ill-health and the RTW process. Returning workers can face challenges in protecting themselves from “overdoing it” and exceeding their current capacity due to difficulties in setting limits in demanding workplace situations (Noordik et al., 2011).

When RTW stakeholders such as supervisors, co-workers, and RTW coordinators have strong mental health literacy they reduce the mental and social suffering of returning workers by offering understanding, empathy and support for psychological wellbeing. In a RTW context, supervisors and other stakeholders need to be aware of the psychological impacts of pain and disability, especially those resulting from prolonged periods of ill-health (Dembe, 2001). They should also understand the psychological impacts of physical, mental, and social suffering the worker has experienced, especially as these relate to being forced by their ill-health (however temporarily) to adopt less valuable roles in their workplaces, families, and communities (MacEachen et al., 2007). This can be achieved by creating opportunities for workers to debrief about their experiences and suffering when planning their RTW planning and re-engaging with the workplace. Support for psychological wellbeing can also be enhanced by providing access to (and time off to attend) counselling support (Kirsh and McKee, 2003), providing mental health first aid training for RTW stakeholders, and actively promoting psychological safety in workplaces.

### Return-to-Work Literacy of Supervisors and Colleagues

The RTW literacy of supervisors and colleagues directly influences the social support or social suffering experienced by returning workers. Work disability is prolonged by low social and practical support from co-workers and supervisors (Krause et al., 2001), and when returning workers are treated with indifference and disrespect by supervisors and co-workers (Gewurtz et al., 2018). Conversely, studies have found that injured workers returning to workplaces in which supervisors were sympathetic were more motivated to persist with challenging tasks and overcome difficulties when dealing with their injuries (Lysaght and Larmour-Trode, 2008).

Return-to-work literacy of supervisors is a crucial influence on the success of the RTW plan and process. Their provision of proactive supervisory support, cooperative relationships with workers, good communication, fairness, and inclusion of the worker in decisions about their work and accommodations are crucial to successful work re-entry by injured workers (Lysaght and Larmour-Trode, 2008). Their capacity to provide emotional support, information support (such as advice on RTW rules and guidelines), instrumental support (including financial support and time) and validation support (through performance feedback) are key influences on the RTW outcomes (Shaw et al., 2009). Indeed, a supervisor’s “experience, knowledge, and support for accommodation[s] can be as important as standard ergonomic principles and medical restrictions” to successful accommodation of worker needs (Shaw et al., 2014, p. 756). Providing this depends, however, on supervisors also knowing how to navigate and accommodate both the worker’s support needs and the organisation’s operational needs.

Co-workers are also important because co-worker support has an even stronger impact on RTW than supervisor support (Haveraaen et al., 2016). Social support from co-workers to returning workers can buffer work stress and psychological strain, reduce work pressure by taking over or reducing the person’s workload (Bostjancic and Koracin, 2014) and helping to improve the returning worker’s resilience, self-confidence, and optimism (Haveraaen et al., 2016). All these supports reduce physical, mental, and social suffering for the returning worker.

Return-to-work literacy of supervisors, co-workers, and other stakeholders can be enhanced by raising awareness of rights, processes and supports mandated by relevant legislation and policy, and that anyone can experience work-related ill-health (Kirsh and McKee, 2003). Education about common workplace injuries and illnesses and their impact on workers enhances RTW literacy by increasing compassion and workplace support for returning workers (Lysaght and Larmour-Trode, 2008).

## Conclusion

Return-to-work is an interactive and fluid process that can either exacerbate or mitigate the physical, mental, existential, and social suffering that ill-health causes workers to experience. The five factors detailed here can mitigate suffering but future studies could explore more fully how each factor influences each form of suffering, how those impacts may differ for different types of worker and workplace contexts, and how they may also differ for workers returning from physical versus psychological ill-health.

## Data Availability Statement

The original contributions presented in the study are included in the article/supplementary material, further inquiries can be directed to the corresponding author.

## Author Contributions

Both authors listed have made a substantial, direct, and intellectual contribution to the work, and approved it for publication.

## Conflict of Interest

The authors declare that the research was conducted in the absence of any commercial or financial relationships that could be construed as a potential conflict of interest.

## Publisher’s Note

All claims expressed in this article are solely those of the authors and do not necessarily represent those of their affiliated organizations, or those of the publisher, the editors and the reviewers. Any product that may be evaluated in this article, or claim that may be made by its manufacturer, is not guaranteed or endorsed by the publisher.
